# Valorization of Onion Waste by Obtaining Extracts Rich in Phenolic Compounds and Feasibility of Its Therapeutic Use on Colon Cancer

**DOI:** 10.3390/antiox11040733

**Published:** 2022-04-07

**Authors:** Mónica Paesa, Danielle Pires Nogueira, Gustavo Velderrain-Rodríguez, Irene Esparza, Nerea Jiménez-Moreno, Gracia Mendoza, Jesús Osada, Olga Martin-Belloso, María Jesús Rodríguez-Yoldi, Carmen Ancín-Azpilicueta

**Affiliations:** 1 Department of Chemical Engineering, University of Zaragoza, Campus Río Ebro-Edificio I+D, C/Poeta Mariano Esquillor S/N, 50018 Zaragoza, Spain; 683886@unizar.es; 2 Instituto de Nanociencia y Materiales de Aragón (INMA), CSIC-Universidad de Zaragoza, 50009 Zaragoza, Spain; 3 Department of Sciences, Institute for Advanced Materials (INAMAT2), Universidad Pública de Navarra, Campus Arrosadía s/n, 31006 Pamplona, Spain; danielle.pires@unavarra.es (D.P.N.); irene.esparza@unavarra.es (I.E.); nerea.jimenez@unavarra.es (N.J.-M.); 4 Alianza Latinoamericana De Nutricion Responsable, Inc., 400 E Randolph St Suite 2305, Chicago, IL 60611, USA; coordinacion.cientifica@alanurla.org; 5 Department of Food Technology, University of Lleida-Agrotecnio Center, Avenue Alcalde Rovira Roure 191, 25198 Lleida, Spain; olga.martin@udl.cat; 6 Aragon Health Research Institute (IIS Aragon), 50009 Zaragoza, Spain; gmendoza@iisaragon.es; 7 Networking Research Center on Bioengineering, Biomaterials and Nanomedicine, CIBER-BBN, 28029 Madrid, Spain; 8 Department of Pharmacology and Physiology, Forensic and Legal Medicine, Veterinary Faculty, University of Zaragoza, 50013 Zaragoza, Spain; 9 CIBERobn, ISCIII, IIS Aragón, IA2, 50009 Zaragoza, Spain; josada@unizar.es; 10 Department of Biochemistry and Molecular and Cellular Biology, Veterinary Faculty, University of Zaragoza, 50013 Zaragoza, Spain

**Keywords:** *Allium cepa* L. by-products, Caco-2 cells, circular economy, intestine, protocatechuic acid, ROS, vegetable extracts

## Abstract

In this study, the total phenolic content, the antioxidant and antiproliferative activities of onion waste extracts were characterized. Some phenolic compounds present in the extracts were also identified and quantified by HPLC-DAD. Additionally, an in-silico analysis was performed to identify the phenolic compounds with the highest intestinal absorption and Caco-2 permeability. The onion extract possessed a high amount of phenolic compounds (177 ± 9 mg/g extract) and had an effective antioxidant capacity measured by ABTS, FRAP and DPPH assays. Regarding the antiproliferative activity, the onion extracts produced cell cycle arrest in the S phase with p53 activation, intrinsic apoptosis (mitochondrial membrane potential modification) and caspase 3 activation. Likewise, onion waste increased intracellular ROS with possible NF-kB activation causing a proteasome down regulation. In addition, the extracts protected the intestine against oxidative stress induced by H_2_O_2_. According to the in-silico analysis, these results could be related to the higher Caco-2 permeability to protocatechuic acid. Therefore, this study provides new insights regarding the potential use of these types of extract as functional ingredients with antioxidant and antiproliferative properties and as medicinal agents in diseases related to oxidative stress, such as cancer. In addition, its valorization would contribute to the circular economy.

## 1. Introduction

The onion (*Allium cepa* L.) bulb is cultivated in all the temperate regions of the world, and is one of the oldest cultivated plants (dating back more than 4000 years) [1]. The genus *Allium* includes more than 750 species distributed in the northern hemisphere. China, USA, India and Japan are the main producing countries. The onion is a plant that requires temperate and warm climates, with a dry environment. Nevertheless, given the large number of existing varieties, it can be easily adapted both to climatic and soil conditions, so it is cultivated and consumed worldwide, being one of the most important world horticultural crops. The interest in this bulb as a food condiment has promoted a growing demand for its production [2]. As a result of this high level of production, large amounts of onion waste and by-products are generated annually in the European Union (more than 500,000 tons of onion waste in 2013) [3]. These residues (that mostly contain the dry skin from peeling, the outer two fleshy leaves and the top and bottom bulbs removed before processing) are not suitable for fodder or landfill disposal [4], however, they are rich in flavonoids [5]. In fact, the concentration levels of these compounds in the edible parts of onions range between 0.03 and 1 g/kg, while it is possible to find up to 10 g/kg in the skins [6]. Therefore, the valorization of onion waste is good from an environmental point of view and could result in great interest for obtaining biomolecules with a high antioxidant potential. Thus, the implementation of economic and scalable valorization methodologies could alleviate the negative consequences derived from the accumulation of these byproducts, and also provide an economic benefit for both the onion producers and processors [4].

Onions contribute significantly to the human diet and, thanks to their bioactive compounds, they have also been used for disease prevention [7,8,9]. The healthy properties traditionally attributed to the onion are mainly due to its high content of flavonols, with more than 25 different ones being identified, among which the most relevant are quercetin and its derivatives [10] along with other compounds such as kaempferol, isorhamnetin and myricetin [11]. In particular, quercetin is one of the most abundant flavonoids found in a variety of plant-based foods and is particularly abundant in red onions (0.3 mg/g fresh weight) [12]. The high content of quercetin and derivatives in onions leads us to think that the onion waste will also have significant concentrations of these bioactive compounds. This is relevant as quercetin and its derivatives are known by its ability to provide an important defense against oxidative stress from oxidizing agents and free radicals [13,14], leading a large number of health benefits, such as cancer-prevention, anti-tumor, anti-microbial and anti-asthmatic, among others [15,16,17].

In these regards, quercetin has been shown to exhibit anticancer activity against different human cancer cell lines, including breast, colorectal, stomach, head and neck, lung, ovarian, melanoma and leukemia through multiple mechanisms of action [18,19,20,21,22]. This flavonol is also reported to arrest the S phase in human breast cancer cells due to an increase in active p53 and p57 proteins [23]. In addition, taxifolin (dihydroquercetin) also presents anticancer activity, and this has been evaluated by both in vitro and in vivo models [15]. The specific chemical composition of the onion, and therefore of its by-products, depends on varieties and the geographical area of cultivation. In this regard, Metrani et al. [24] found different content in flavonoids, phenolic compounds, anthocyanins and proteins in onions from different origins. In addition, storage, processing and post-harvest treatments of onions could modify the structure of some bioactive compounds by altering their bioavailability [25].

In view of all of this, the aim of the present work is to assess the therapeutic potential in both the prevention and treatment of colon cancer of an extract obtained from Spanish onion wastes by solid-liquid extraction. Cell experiments represent the first step in evaluating the health effect of these extracts for use as new functional ingredients.

This study is part of a project that we have been developing in our laboratory about plant extracts and gold metal complex applications on colon cancer.

## 2. Material and Methods

### 2.1. Extracts

The onion waste, composed of the peels and top and bottom bulbs from Spanish onions (from Castilla-La Mancha, Spain), were collected from household waste. Firstly, samples were dried at 30 °C until a constant weight. Then, dried samples were milled with a coffee grinder so all the material could be sieved through a 300 µm sieve. The extractions were conducted with Erlenmeyer flasks where 1 g of onion waste powder and 100 mL of ethanol:water (50:50 v:v) were added. Said flasks were put in an orbital shaker (250 rpm) inside a stove (Ing Climas, Barcelona, Spain) at 40 °C for 24 h. After this time, the extraction solution was centrifuged for 15 min at 8000 rpm (which corresponds to a relative centrifugal force of 8586× *g*) and filtered. The filtered liquid was then rotoevaporated and frozen at −20 °C before lyophilization. Finally, it was lyophilized in a Telstar Cryodos freeze-drier (Madrid, Spain). The extraction yield was 20%. The freeze-dried extract was stored under refrigeration in clear glass vials.

### 2.2. Characterization of Phenolic Compounds of Onions Extracts by HPLC-DAD

The quantification of phenolic compounds in onion extracts were performed by using HPLC-DAD. For this, 30 mg of freeze-dried extract were reconstituted in 350 µL of methanol, and filtered with a 0.45 µm PTFE syringe filter. The sample preparation procedure was conducted in triplicate. The samples were analyzed using a high-performance liquid chromatograph equipped with two 510 pumps, a 717 Plus autosampler, a 996 photodiode array detector (Waters Div., Milford, MA, USA). A Luna reversed phase LC column (250 mm × 4.6 mm, 5 µm) (Phenomenex, Torrance, CA, USA) was used. The chromatographic analyses were carried out according to a modified method [26]. Briefly, the mobile phases were 0.5% formic acid in water (A) and 0.5% formic acid in acetonitrile (B). The gradient of A was: 95% (0–5 min); 95–82% (5–30 min); 82–30% (30–90 min); 30–10% (90–100 min); 10–95% (100–120 min). The flow rate was 0.8 mL/min, the oven temperature 30 °C, and the injection volume 10 µL. In order to identify the largest number of phenolic compounds, commercial standards of the following compounds were injected: gallic acid, ellagic acid, protocatechuic acid, chlorogenic acid, neochlorogenic acid, caftaric acid, vanillic acid, caffeic acid, syringic acid, cinnamic acid, *p*-coumaric acid, ferulic acid, procyanidin B1, catechin, epicatechin, malvidin-3-glucoside, polydatin, quercetin, quercetin-3-glucoside, quercetin-3-glucuronide, quercetin 3,4’-diglucoside, kaempferol, isorhamnetin, piceatannol, resveratrol, and viniferin. All the HPLC quality standards were from Sigma-Aldrich (Madrid, Spain), with the exception of procyanidin B1, malvidin-3-glucoside, quercetin-3-glucuronide, quercetin 3,4’-diglucoside, isorhamnetin and viniferin (Oenyn chloride, Extrasynthese, Genay, France) and polydatin (PhytoLab GmbH and Co, Vestenbergsgreuth, Germany). Identification of the different compounds was conducted by the double coincidence of the retention time of the corresponding standard and the UV spectrum. Quantification was carried out through a calibration curve for each of the compounds. All the calibration curves used for the analysis presented determination coefficients higher than 0.998. Results of the extract composition were expressed as mg/g of dry extract.

### 2.3. Antioxidant Capacity of Onion Extract by DPPH, ABTS and FRAP

The antioxidant capacity of the extracts was determined with three different spectrophotometric methods, by using a UV-Vis spectrophotometer (Jenway 7315, Staffordshire, UK). The DPPH (2,2-diphenyl-1-pycrilhydracyl) assay was conducted following the method previously described [27]. A total of three different processed samples were prepared, and each of them was analyzed once. For sample preparation, between 49.1 ± 0.1 mg and 53.1 ± 0.1 mg of the extract were dissolved in 5 mL of methanol and the resulting solution was then filtered through a PTFE filter and diluted 10 times with methanol. For the calibration curve, Trolox was used as standard with a concentration range of 0.05 to 0.56 mM. The ABTS (2,2′-azinobis(3-ethylbenzothiazoline-6-sulphonic acid)) method used was originally described in [28]. The calibration curve was also prepared with Trolox as standard, and the concentration range varied from 0.05 to 1.2 mM. The antioxidant activity of the different extracts was also measured by the FRAP method [28]. Known concentrations of Trolox, in the range of 0.05 to 0.5 mM were used for preparing the calibration curve. The determination coefficient obtained for the calibration curves was *R*^2^ = 0.999 in all cases. Results of antioxidant activity were expressed as mmol Trolox equivalents (TE)/g of dry extract.

### 2.4. Determination of Total Phenolic and Total Flavonoids Content

First, 50 mg of freeze-dried extract were reconstituted in 5 mL of methanol, and filtered with a 0.45 µm PTFE syringe filter. Then, samples were diluted four times with methanol for total phenolic quantification, and 30 times for total flavonoid determination. The sample preparation procedure was conducted in triplicate in all cases.

The total polyphenol content was determined by the Folin–Ciocalteu method [29]. Briefly, 7.9 mL of deionized water and 0.5 mL of the Folin–Ciocalteu reagent were added to 0.1 mL of each extract sample. The mixtures were incubated for 2 min, and then 1.5 mL of 20% Na_2_CO_3_ were added. After 2 h in darkness, the absorbance of the samples was measured at 765 nm. A calibration curve with gallic acid concentrations between 0.2 and 5.1 mM was used for the quantification (*R*^2^ = 0.999). Results were expressed as mg of gallic acid equivalents per gram of dry extract.

The total flavonoid content was assessed through a colorimetric method based on the reaction of flavonoids with aluminum chloride in acetic acid [30]. For the sample analysis, 1.5 mL of onion extract solution was mixed with 1.5 mL of a 2% AlCl_3_ solution (prepared in 5% acetic acid), and the resulting solutions were left for 30 min in darkness. For flavonoid quantification, a calibration curve between 3.0 and 30.0 µg/mL of quercetin was prepared (*R*^2^ = 0.9994). Absorbance was measured at 420 nm. Results were expressed as mg of quercetin equivalents per gram of dry extract.

### 2.5. Biological Studies

Human Caco-2 cell line (TC7 clone) was kindly provided by Dr. Edith Brot-Laroche (Université Pierre et Marie Curie-Paris 6, UMR S 872, Les Cordeliers, Paris, France). The cell line was maintained in a humidified atmosphere of 5% CO_2_ at 37 °C. Cells were cultured in Dulbecco’s Modified Eagle’s Medium (DMEM) (Biowest, Nuaillé, France) containing 2 mM L-glutamine supplemented with 10% fetal bovine serum (FBS), 1% non-essential amino acids, and a mixture of 1% antibiotics and amphotericin (Biowest, France). Cells were trypsinized with 0.25% trypsin-1 mM and subcultured in 25 cm^2^ plastic flasks at a density of 3 × 10^5^ cells/cm^2^. The culture medium was changed every 2–3 days. The extracts were added to the cells 24 h after seeding in the case of undifferentiated Caco-2 cells and at 25 days in the case of differentiated cells. Cell confluence (80%) was determined by light microscopy.

#### 2.5.1. Cell Proliferation Assay and IC_50_Values

Extracts from the onion waste were diluted in a supplemented cell culture medium to the final concentration of 1 mg/mL. For cytotoxicity screening assays, the cells were seeded in 96-well plates at a density of 4 × 10^3^ cells/well. The culture medium was replaced with a medium containing onion extracts and the cells were incubated for 24, 48 and 72 h. The cells were washed twice with PBS after treatment and the Blue Cell Viability Assay (Abnova, Taipei, Taiwan) was developed by adding 10% of the reagent to the supplemented medium. After 3 h of incubation, viability was evaluated by fluorescence reading in a microplate reader (Multimode Synergy HT Microplate Reader; Biotek, Shoreline, WA 98133, USA) at 530 nm excitation and 590 nm emission wavelengths. Cell viability was calculated by linear interpolation of the fluorescence data from the treated cells versus the not treated sample (control sample containing 1% ethanol, 100% viability). The IC_50_ value indicates the concentration of the compound is capable of reducing cell viability to 50%. This value was calculated in all conditions tested and was chosen for subsequent assays.

#### 2.5.2. Propidium Iodide Staining of DNA Content and Cell Cycle Analysis

The onion’s effect on Caco-2 cell cycle was studied by PI/RNase solution kit (Immunostep, Salamanca, Spain). Briefly, cells were fixed in 70% ice-cold ethanol and stored at 4 °C for 48 h. After centrifugation (413× *g*, 5 min), cells were rehydrated in PBS and stained with propidium iodide (PI) solution (50 μg/mL) containing RNase A (100 μg/mL). PI-stained cells were analyzed for DNA content by flow cytometry using a Beckman Coulter Gallios cytometer (Brea, CA, USA).

#### 2.5.3. Measurements of Apoptosis

The cells were seeded in 25 cm^2^ flasks (3 × 10^5^ cells/cm^2^) and then exposed to onion extract for 48 h at IC_50_ concentration, then collected and stained with annexin V-FITC and propidium iodide as previously described [31]. A negative control was prepared by untreated cells, that was used to define the basal level of apoptotic and necrotic or dead cells. After incubation, cells were transferred to flow cytometry tubes and washed twice with phosphate saline buffer (PBS), followed by a resuspension in 100 µL of annexing V binding buffer (100 mM Hepes/NaOH pH 7.4, 140 mM NaCl, 2.5 mM CaCl_2_). Then, 5 µL of Annexin V-FITC and 5 µL of propidium iodide were added to each tube. After 15 min of incubation at room temperature in the dark, 400 µL of annexin binding buffer was added. The signal intensity was measured by flow cytometry using a Beckman Coulter Gallios (Brea, CA, USA). Apoptosis values are expressed as a percentage.

#### 2.5.4. Mitochondrial Membrane Potential Assay

The assay was performed by flow cytometry. For this, the cells were seeded in 25 cm^2^ flasks and treated with onion extracts for 48 h. The extract-free medium was added to the control cells. After the treatment time, the cells were washed twice with PBS and resuspended in PBS at concentration of 10^5^ cell/mL and 5 μL of 10 μM 1,1′,3,3,3′-hexamethylindodicarbo-cyanine iodide (DiIC1) were added to each sample. Tubes were incubated at 37 °C for 15 min and 400 μL of PBS was added prior to the analysis of fluorescence with a Beckman Coulter Gallios flow cytometer (Brea, CA, USA). Excitation and emission settings were 633 and 658 nm, respectively [31]. Values are expressed as a percentage of cells with a changed mitochondrial potential.

#### 2.5.5. Determination of P53 and Caspase 3 Activity

Caco-2 cells were plated in 25 cm^2^ flasks at a density of 3 × 10^5^ cells per flask and incubated for 24 h under standard cell culture conditions. Then, 1000 μg/mL onion solution was added to the samples and incubated for 48 h. Then cells were collected and processed following instructions by Sánchez de Diego et al. [31]. Finally, 100 µL of every sample was incubated with 5 µL p53 antibody (1:20) (Miltenyi, Cologne, Germany, Clone REA609) and 5 µL of anti-active caspase-3 (1:20) (BD Pharmigen, Clone C92-605). Fluorescence was measured by flow cytometry using a Beckman Coulter Gallios cytometer (Brea, CA, USA) equipped with a blue solid diode laser (488 nm) and a red solid diode laser (635 nm). For p53 analysis excitation at 635 nm and emission at 660 nm and for caspase-3 determination excitation and emission wavelengths were set at 488 and 525 nm, respectively. Values are expressed as a percentage of cells with active protein.

#### 2.5.6. Determination of Proteasome Activity

Caco-2 cells (8 × 10^4^ cells/well/90 μL) were seeded in black 96-well plates with growth medium overnight. The determination of the proteasome activity was carried out by fluorometric assay using a proteasome 20S activity assay kit (MAK172, Sigma-Aldrich) based on Suc-LLVy-AMC, a fluorogenic substrate of the proteasome β5 submit. Caco-2 cells were treated with 10 μL of 1000 μg/mL onion extracts for 48 h and then the cells were processed following the instructions in the kit protocol. The fluorescence levels correspond to the proteasomal chymotrypsin-like activity (CT-L activity). Increasing fluorescence intensity indicates increased proteasome 20S activity. The activity was measured in lysed cells with FLUOstar Omega (BMG Labtech, Ortenberg, Germany) and the value was obtained per mg of protein. The data are expressed as fluorescence intensity.

#### 2.5.7. Determination of Intracellular Levels of Reactive Oxygen Species (ROS)

The cells were seeded in 96-wells plate at a density of 4 × 10^3^ cells/well. The intracellular level of ROS was assessed using the dichlorofluorescein assay as previously described [31]. Cells were cultured before oxidative stress induction, and then incubated with onion extract for 24 and 48 h. After that, the medium was removed, cells were washed twice with phosphate buffered saline, and incubated for 20 min with 20 μM 2′,7′–dichlorofluorescein diacetate (DCFH-DA) in PBS at 37 °C. The formation of the fluorescence oxidized derivative of DCF was monitored in a multiplate reader at emission and excitation wavelengths of 535 and 485 nm, respectively. A measure at time “zero” was also performed. Cells were then incubated at 37 °C in the multiplate reader, and the generation of fluorescence was measured after 20 min. The ROS levels were expressed as a percentage of fluorescence compared to the control. The obtained values of fluorescence intensity are considered as a reflection of the total intracellular reactive oxygen species content.

#### 2.5.8. Theoretical Absorption Percentage and Caco-2 Permeability of Individual Phenolic Compounds 

The theoretical absorption percentage and Caco-2 permeability of the main individual phenolic compounds found in onion waste were evaluated as described by Velderrain-Rodríguez et al. [32]. Briefly, the theoretical absorption was simulated by using the chemical structures, SMILES (simplified molecular-input line-entry system) codes of the phenolic compounds and the “Molinspiration online property calculation toolkit” (http://www.molinspiration.com/, accessed on 2 March 2022). The SMILES codes were taken from the PubChem Open Chemistry Database (https://pubchem.ncbi.nlm.nih.gov/search/, accessed on 2 March 2022). As for the Caco-2 permeability, it was predicted using the online program pkCSM (http://biosig.unimelb.edu.au/pkcsm/prediction, accessed on 2 March 2022), which is based on 674 drug like molecules with Caco-2 permeability values and predicts the logarithm of the apparent permeability coefficient (log P_app_). Thus, the Caco-2 permeability of the phenolic compounds from onion waste are displayed as the log P_app_ and expressed as 10^−6^ cm/s.

### 2.6. Statistical Analysis

Statistical analysis of the data was performed using GraphPad version 7.04 software (GraphPad Software, Inc. La Jolla, CA, USA) on a PC computer. A one-way analysis of variance (ANOVA) set was used for multiple comparisons with a Dunnett’s post-test and an unpaired t-test. All tests were performed at least three times. Data are presented as mean ± SD.

## 3. Results and Discussion

### 3.1. Phenolic Composition and Antioxidant Activity in Onion WasteExtracts

In total, seven different phenolic compounds were unambiguously identified in the onion waste extract obtained from onion waste in comparison (spectrum and retention time) to the corresponding reference standards. Three of them (protocatechuic acid, vanillic acid and ellagic acid) are phenolic acids, and the rest are flavonols, with quercetin being one of the main components. 

**Figure 1:**
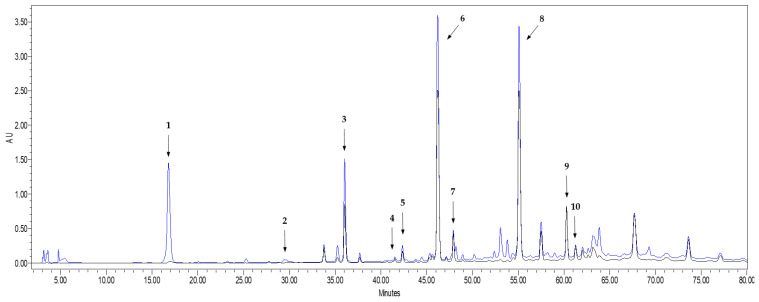
HPLC-UV chromatogram recorded at 254 nm (blue line) and 365 nm (black line) of the extract obtained from onion waste. Peak 1: Protocatechuic acid; Peak 2: Vanillic acid; Peak 3: Unknown flavonoid 1; Peak 4: Ellagic acid; Peak 5: Quercetin 3-glucoside; Peak 6: Unknown flavonoid 2; Peak 7: Unknown flavonoid 3; Peak 8: Quercetin; Peak 9: Kaempferol; Peak 10: Isorhamnetin.

As it can be seen in Figure 1, all the flavonoid compounds identified in the chromatogram present high absorbance values at both 254 nm and 365 nm, while the phenolic acids did not absorb at 365 nm. This is in agreement with Lee et al. [26], who explained that UV-visible spectra of flavonoids present two absorption maxima: one of them in the 352–385 nm region and the other in the 205–260 nm region. Besides the identified peaks, Figure 1 shows three additional chromatographic peaks whose spectrum fit well with a flavonoid compound. By comparing with bibliographic spectra [26,33], the UV-spectra of the unknown compound 1 (peak 3 in Figure 1) could be identified as quercetin 3,7,4′-triglucoside, or isorhamnetin 3,4′-diglucoside, while the unknown compounds 2 and 3 (peaks 6 and 7 in Figure 1) could correspond to quercetin 4′-glucoside, isorhamnetin 4′-glucoside or quercetin 7,4′-diglucoside (see UV spectra of the unidentified peaks in Figure S1 of the Supplementary Material). Unfortunately, the unavailability of standards of such quercetin derivatives did not allow us to unambiguously identify the three compounds. However, they were considered as quercetin derivatives and quantified as quercetin equivalents, since their spectral similarities with the spectrum of quercetin makes their determination relatively feasible as “external standard quercetin equivalents” [4].

Table 1 shows the concentration values (determined by HPLC-DAD and expressed as mg/g dry extract) of the different polyphenols found in the extracts. The presence of the identified acids and flavonols in onion extracts have also been reported in onion by-products by other authors [4,5,34]. A comparison of the concentration values obtained for the different compounds with results obtained by other research works is very difficult, since the onion wastes and extraction protocols are different, and also data are presented in different units. However, most researchers identified quercetin and its derivatives as the main component, which agrees with the results obtained in the present work.

In addition to the individual phenolic compounds, the antioxidant capacity of the extracts was also determined (Table 2). For that purpose, ABTS, DPPH and FRAP assays were applied in order to have a full view on the antioxidant potential of the extracts, since its complex nature makes analysis by a single method difficult. As can be seen, the values of antioxidant capacity obtained for the three methods were different from each other, suggesting that these molecules may be acting on different antioxidant mechanisms according to the free radical structure and its chemical environment. In that sense, the highest antioxidant capacity values were obtained by the ABTS method, and the lowest by the DPPH one. This is in agreement with Campone et al. [4], who also found higher antioxidant capacity values with the ABTS assay than with the DPPH test when they analyzed extracts obtained from onion wastes. On the other hand, Ren et al. [9] found lower antioxidant capacity values for onion extracts measured by DPPH assay than those obtained from a FRAP test.

With regard to the total phenolic and total flavonoid content found in our extracts (Table 2), the values obtained in the present study were higher than those obtained by Nile et al. [35] from red onion solid wastes extracted with distilled water. However, when this author used ethanol 80% as an extraction solvent, both total phenolic and total flavonoid values were higher than the results obtained in the present study. Therefore, these differences could be attributed to the different extraction protocol used, although the different onion variety could also influence such differences.

### 3.2. Biological Studies with OnionWasteExtract

Natural compounds are promising sources for the development of new remedies for different diseases and it has been estimated that numerous types of cancer can be prevented by a healthy lifestyle [36]. Many studies established that polyphenols compounds have positive influence on health, such as antidiabetic, antiviral, antioxidant, anti-inflammatory, and anticancer effects [27].

The anticancer potential of polyphenols was well addressed, most probably due to their wide distribution in nature and versatile chemical characters [37]. This includes a large number of polyphenol-rich extracts from plants as well as isolated pure compounds [38], and thus, covers almost all types of cancers including multiple drug resistant cases [39]. Likewise, polyphenols showed strong in vivo and in vitro anti-proliferative effects through decreasing cell proliferation, the inactivation of carcinogens, angiogenesis inhibition, the induction of cell cycle arrest and apoptosis and modulating immunity on various human cancer cells with few or no toxicity effects on normal cells [40,41,42].

#### 3.2.1. Antiproliferative Effect and Cell Cycle Arrest

**Figure 2:**
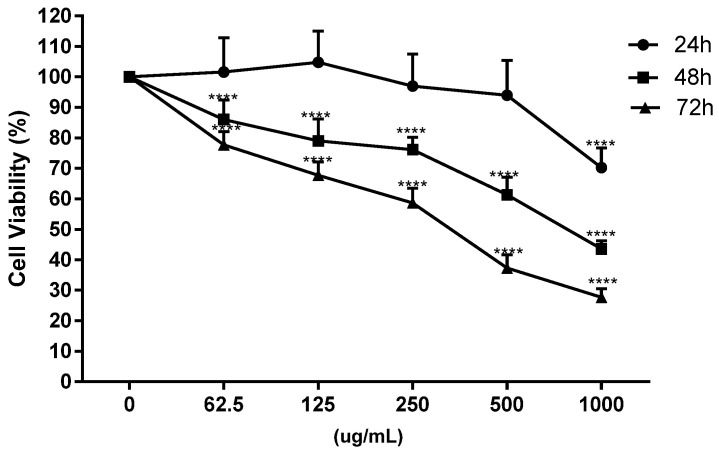
Dose-response curve of cell viability in Caco-2 cells at different incubation times (24, 48 and 72 h) and concentrations (0, 62.5, 125, 250, 500 and 1000 μg/mL) with onion waste extracts (**** *p* < 0.0001).

On undifferentiated Caco-2 cells, the toxicity of onion extracts was evaluated at different concentrations (62.5, 125, 250, 500 and 1000 μg/mL) and times (24, 48 and 72 h) in relation to previous studies carried out in our laboratory with other plant extracts [32,43] and the IC_50_ at 48 h was chosen for next assays (Figure 2). 

**Figure 3:**
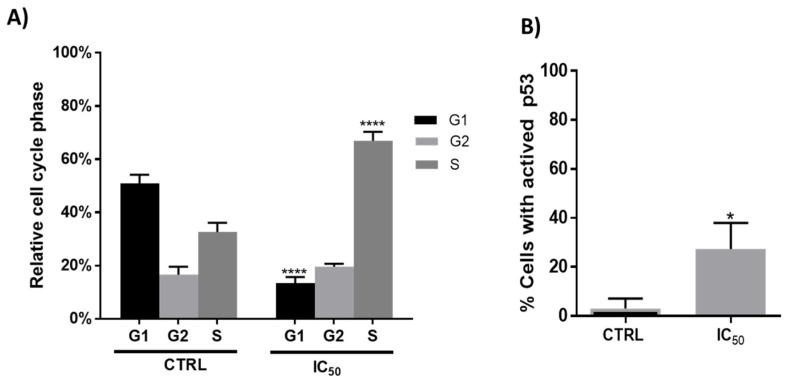
(**A**). Relative cell cycle phases in Caco-2 cells after 48 h incubation in absence (CTRL) or presence (IC_50_ = 1000 μg/mL) of onion extracts **** *p* < 0.0001. (**B**). Percentage of Caco-2 cells with presence of active p53 after 48 h incubation with onion extract at IC_50_ (1000 μg/mL). * *p* < 0.05 vs. CTRL (untreated cells).

Cell death is often accompanied by cell cycle disturbances, and previous studies with plant extracts suggested that treated cells can arrest progression at different phases of the cell cycle [32,43]. In the present study, flow cytometry analysis showed that after 48 h of incubation, the onion extracts, at 1000 μg/mL, altered the cell cycle initiation displaying a significant arrest in the S phase with a concomitant decrease in the G1 phase compared to untreated cells (Figure 3, panel A). These results agree with those obtained by Chou et al. in human breast cancer MCF-7 cells [23].

One of the major genes that have an influence on the regulation of the cell cycle (progression of cell division) is the tumor suppressor gene p53 [44,45]. The activation of the p53 protein is regulated at the molecular level by several covalent modifications including acetylation, methylation, ubiquitination and phosphorylation [46]. It is also well known that the anticancer effect of phenolic compounds is attributed to multiple mechanisms such as induction of apoptosis, regulation of various signaling pathways, regulation of cell cycle, and activation of receptors at the level of plasma membrane [47]. In fact, the induction of the p53 protein by phenolic compounds such as quercetin has been documented [23,36,48,49,50]. As shown in Figure 3B, the incubation with onion extracts showed an increase in the number of cells with p53 activated with respect to the untreated cells. 

#### 3.2.2. Type of Cell Death

Apoptosis or programmed cell death is defined as an active physiological process of cell self-destruction, with specific morphological and biochemical changes in the nucleus and cytoplasm. Apoptotic death is known to involve a cascade of proteolytic events driven mainly by a family of cysteine proteases such as caspase-3, the major executioner of apoptosis. Agents that suppress the proliferation of malignant cells, by inducing apoptosis, may represent a useful mechanistic approach to both chemoprevention and chemotherapy of cancer.

Cancer cells often suppress the p53 protein, up regulating anti-apoptotic Bcl 2 family proteins. The suppression of p53 also results in the inhibition of caspase enzymes, such as caspase 3 [51]. Furthermore, p53 can induce apoptosis cell death in response to DNA damage [52]. Thus, they are known to primarily target the p53 signaling pathway to produce an anticancer activity through apoptosis in a variety of cancers [53].

**Figure 4:**
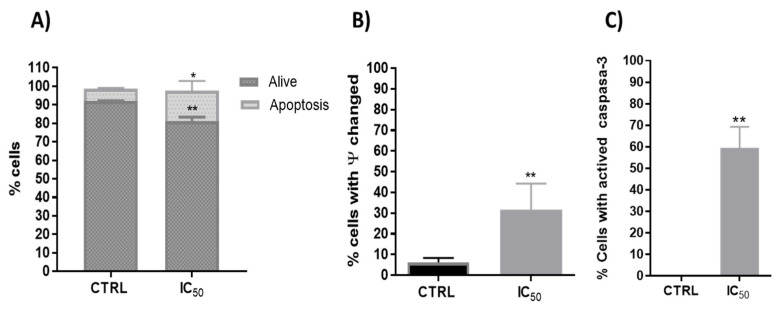
(**A**). Percentage of the type of cell death induced on Caco-2 cells after 48 h incubation in CTRL (untreated cells) and onion extract at IC_50_ (1000 μg/mL) * *p* < 0.05; ** *p* < 0.01. (**B**). Analysis of mitochondrial membrane potential (∆ψm) after 48 h incubation with onion extract at IC_50_ ** *p* < 0.01 vs. CTRL. (**C**). Percentage of Caco-2 cells with presence of active caspase-3 after 48 h incubation with onion extract at IC_50_ ** *p* < 0.01 vs. CTRL.

Phenolic compounds exhibited potential anticancer activity through numerous signaling pathways, including the death receptor (extrinsic) pathway, the mitochondrial (intrinsic) pathway and the perforin/granzyme apoptotic pathway [54]. Polyphenols used the p53 signaling pathway to produce anticancer activity through apoptosis in a variety of cancers [55,56]. Thus, one investigation evidenced that quercetin upregulated p53 expression and decreased the expression of MDM2 in resistant transformed follicular lymphoma B-cell lines [57] and in breast cancer MCF-7 cells [23]. In this way, the onion extracts showed a significant increase in apoptosis on Caco-2 cells (Figure 4A).

In addition, one of the first steps leading to apoptosis is mitochondrial depolarization. Throughout the apoptotic process, the mitochondrion undergoes the redistribution of hydrogen ions, altering its membrane potential that leads to the release of cytochrome c to the cytoplasm and the activation of caspase-3, which executes the apoptosis. Therefore, the onion extracts effect on the mitochondrial membrane potential and caspase 3 activity were analyzed. In this way, previous studies with plant extracts have shown mitochondrial dysfunction and intrinsic apoptosis induction [32,43]. Flow cytometry determination showed that onion extracts significantly alter the number of cells with potential mitochondrial changes (Figure 4B) and activated caspase-3 (Figure 4C) that would favor the apoptosis found caused by onion extracts (Figure 4A). Therefore, cell death or decreased viability could have been induced, at least in part, by mitochondrial pathway with activation of p53 and caspase 3. These events cause cell cycle arrest, as p53 has been shown to greatly influence the cell cycle progression [58].

#### 3.2.3. Activity of Proteasome 20 S Subunit

The main proteolytic pathway in eukaryotic cells is the ubiquitin/proteasome system (UPS). The UPS participates in the regulation of biological processes such as cell growth, proliferation, cell cycle and apoptosis [59] and the deregulation of these processes causes the cells to become malignant. Therefore, several cancer cells have UPS dysfunction with increased proteasome activity [60]. Some studies have shown that proteasome inhibition in cancer cells can lead to the accumulation of cyclin-dependent kinase inhibitors, proapoptotics, and tumor suppressor proteins, leading to apoptosis [61]. The nucleus of the proteasome presents the 20S subunit, involved in the activation of the NF-kB factor. This factor in the cytoplasm is associated with inhibitory proteins known as IkBs, and its activation involves the phosphorylation, ubiquitinization, and degradation of IkB by the proteasome. Once the NF-kB factor is released, it translocates to the nucleus where it binds to DNA and induces the transcription of different mediators.

Some studies show the interaction between the p53 protein and the NF-kB pathway through polyphenols. Thus, the flavonoid fisetin produces an inhibition of bladder cancer by activation of p53 and downregulation of NF-kB pathway in a rat bladder carcinogenesis model [62]. Likewise, the antitumorigenic potential of tea polyphenols induced in Wistar rats, lead to the scavenging of ROS by inhibiting cyclooxygenase-2 (Cox-2) and inactivating phosphorylated form of NF-kB and Akt [63]. In this sense, it has been evidenced in DLD1 and LoVo human colon cancer cells that p53 activation reduces the constitutive activity of NF-kB probably by enhancing cytoplasmic IkB expression and contributing to apoptosis [64]. 

**Figure 5:**
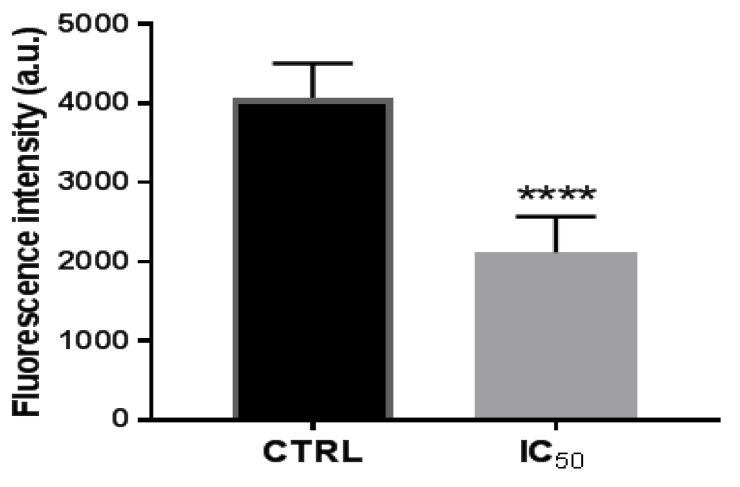
Proteasome 20 S activity in Caco-2 cells after incubation with onion extract at IC_50_ (1000 μg/mL) for 48 h. **** *p* < 0.0001 vs. CTRL (untreated cells).

For all these, it was analyzed whether the onion waste extracts were capable of modifying the activity of the proteasome. The results showed that, after 48 h of treatment of the cells with the extracts, the activity of proteasomal CT-L decreased (Figure 5), perhaps due to NF-kB being downregulated by the activation of the p53 protein, as we have seen in Figure 3B.

#### 3.2.4. Redox Activity on Colon Cancer Cells

**Figure 6:**
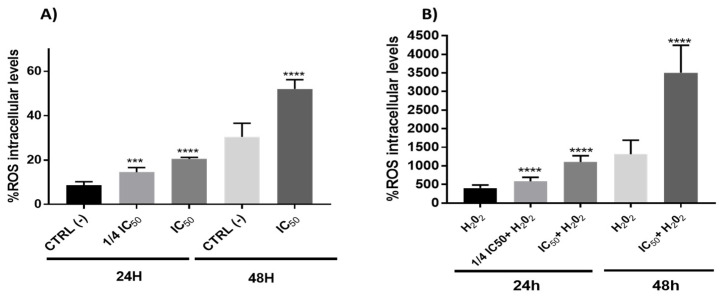
Measurements of ROS levels on Caco-2 cells in absence (**A**) or presence (**B**) of H_2_O_2_ (80 mM, 20 min) after 24 or 48 h incubation with onion extract at IC_50_ (1000 μg/mL) or ¼ IC_50_ (250 μg/mL). *** *p* < 0.001; **** *p* < 0.0001 vs. CTRL (left) or H_2_O_2_ (right).

The interaction of ROS with cellular compounds and their function as cellular messengers to modulate diverse redox-sensitive pathways, including p53 signaling pathway or mitochondrial dysfunction, can be directly implicated in the ethology and progression of cancer [65]. The p53 protein shows both pro-oxidant and antioxidant functions in relation to the intensity of the stress, which can contribute to tumor suppression [66]. In this study, the results showed a clear pro-oxidant effect in both conditions (with/without H_2_O_2_) being dependent on time and the concentration of the onion extracts (Figure 6). On the other hand, ROS often stimulates the NF-kB pathway in the cytoplasm, yet inhibits NF-kB in the nucleus [67]. In cytoplasm, the ROS has been shown to activate NF-kB through alternative phosphorylation of IkBα, which may or not result in the degradation of IkBα [68]. Therefore, the increase in ROS levels (Figure 6) produced by onion extracts could activate the NF-kB pathway in the cytoplasm with a possible IkBα alternative phosphorylation causing a proteasome down regulation. However, we cannot rule out the possibility that this effect could also be due to the action of p53 on the NF-kB factor caused by the extracts through the extrinsic pathway of apoptosis. In addition, although the antioxidant effect of polyphenols has been extensively studied [27], they may also have pro-oxidant effects. These results have been mainly observed in tumor cells and have been related to the pro-apoptotic action. The dual pro-oxidant and antioxidant behavior of phenolic plant compounds not only depends on the cell type but also on their concentration, chemical structure and pH status [69,70].

#### 3.2.5. Redox Activity on a Model of Intestinal Barrier

Phenolic compounds have an important role in the prevention of gastrointestinal diseases related to oxidative stress [71,72]. The origin of pro-oxidant agents in the intestine is very varied, from drugs and compounds present in food to intestinal pathogens [73,74]. In this context, dietary intervention with antioxidant supplementation has been suggested as a new therapeutic approach to reduce free radical-related intestinal barrier damage. Thus, Catanzaro et al. [75] found that differentiated Caco-2 cells incubated with *Boswellia serrata* extracts were protected against H_2_O_2_-induced damage.

Considering the antioxidant capacity and the phenolic content of the onion waste extracts (Table 1 and Table 2), and the lack of effect on cancer cells, it seemed interesting to evaluate whether or not these extracts showed antioxidant capacity on a model of the intestinal barrier (differentiated Caco-2 cells) upon exogenous oxidative stress by hydrogen peroxide insult or in its absence. In addition, it seemed interesting to test the ability of onion extracts to protect normal cells from oxidative stress, reducing ROS levels in order to elucidate whether these extracts would be useful not only in cancer treatment, but also in prevention of cancer onset. This cell line spontaneously acquires the phenotypic characteristics of noncancerous enterocytes after reaching confluence (differentiated cells). Caco-2 monolayer cells form tight junctions and exhibit the cylindrical polarized morphology of enterocytes, expressing functional microvilli on the apical membrane [76]. Therefore, differentiated Caco-2 cells have been established as an acceptable model of intestinal barrier in vitro [77].

**Figure 7:**
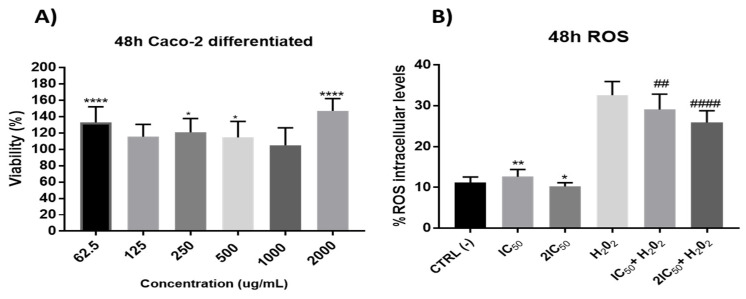
(**A**) Measurement of cell viability in differentiated Caco-2 cells after incubation with onion extracts at 62.5, 125, 250, 500, 1000 and 2000 μg/mL for 48 h. * *p* < 0.5; **** *p* < 0.0001. (**B**). Measurements of ROS levels on differentiated Caco-2 cells in absence or presence of H_2_O_2_ (80 mM, 20 min) after 48 h of incubation with onion extract at IC_50_ (1000 µg/mL) and 2xIC_50_ (2000 µg/mL). ## *p* < 0.01; #### *p* < 0.0001 vs. H_2_O_2_.; * *p* < 0.05; ** *p* < 0.01 vs. CTRL.

Firstly, in these differentiated cells, the possible effect *per se* of the extracts was studied. The results did not show significant reduction in cell viability (Figure 7), which led us to study its possible protective role against oxidative stress. With this purpose, the antioxidant capacity after 48 h incubation time of onion extracts was tested at the IC_50_ and 2xIC_50_ concentrations obtained from undifferentiated cells. The results showed a significant antioxidant effect by preventing H_2_O_2_-induced ROS production without presenting a clear alteration of the redox balance in situations free of oxidative stress (without H_2_O_2_) (Figure 7).

The antioxidant capacity of plant extracts is strongly correlated with its clinical application in gastrointestinal diseases related to oxidative stress [71,72]. These results obtained with onion extracts suggest that they could have a potential application in the management of gastrointestinal diseases related to oxidative stress.

### 3.3. Theoretical Absorption of Individual Phenolic Compounds (Based on Lipinnski Parameters)

The theoretical research approach has been incorporated into experimental studies to support the geometrical analysis, molecular interactions, antioxidant properties, and optical properties of diverse compounds. Theoretical models allow us to explain and understand the behavior of the observed phenomena at a molecular level, and thus, we can convert in silico studies into an important tool that provides significant information about the bioavailability and bioactivity of functional ingredients, such as phenolic compounds. In that sense, the molecular properties of the phenolic compounds found in onion waste extracts are depicted in Table 3. According to these results, the highest theoretical absorption was observed for the phenolic acids of onion waste extracts; these being protocatechuic acid as the phenolic compound with the highest absorption (89.15%), followed by vanillic acid (85.97%) and ellagic acid (60.24%). As for the onion waste flavonoids, the highest theoretical absorption was observed for kaempferol (70.66%). Moreover, quercetin and isorhamnetin had a similar absorption value (63.68% and 67.48, respectively) whereas the glucose molecule of quercetin 3-glucoside significantly reduces its absorption value (36.38%). Lastly, considering the possible matches for the unknown flavonoids’ spectra, all the quercetin and isorhamnetin derivatives had lower absorption values. The reduced absorption of the glycosidic molecules could be related to either an increased total polar surface area (TPSA), the higher molecular weight or the increase in their molecule rotatable bonds.

In addition to the theoretical absorption percentage of the individual phenolic compounds, the Caco-2 permeability prediction could be also used to support these results. The Caco-2 monolayer of cells are widely used as an in vitro model of the human intestinal mucosa and have been showing interesting results that predict the absorption of bioactive molecules. Thus, using the pkCSM predictive model, we may consider that a molecule may display a high Caco-2 permeability if it has log P_app_ value higher than 0.90 × 10^−6^ cm/s [78]. In that sense, protocatechuic acid is the phenolic compound in onion waste extracts with the highest P_app_ value (1.15 × 10^−6^ cm/s), and thus, with the highest Caco-2 permeability. These results agree with the absorption percentage previously discussed for protocatechuic acid. The Caco-2 permeability of protocatechuic acid is comparable to that for other phenolic acids such as vanillin (1.21 × 10^−6^ cm/s), hydroxytyrosol (1.09 × 10^−6^ cm/s) and *p*-coumaric acid (1.21 × 10^−6^ cm/s) as reported by Velderrain-Rodríguez et al. [32]. In that sense, the results of this study suggest that the antiproliferative activity of onion waste extracts observed in this study may be closely related to the phenolic acids and flavonoids with higher absorption and permeability values.

## 4. Conclusions

In this work, we focused our attention on the phenolic compounds present in extracts from onion waste and their application for therapeutic purposes. The extracts have been tested both in human colon cancer cells and in normal enterocytes. Cancer studies showed that onion extracts are capable of decreasing cell proliferation by arresting the cell cycle in S phase, showing a significant increase in the p53 protein activity involved in this process. Likewise, death by the intrinsic apoptosis of cells with the activation of caspase 3 was determined. The extracts produced a pro-oxidant effect in Caco-2 cells with the possible activation of the cytoplasmic NF-kB factor causing a proteasome down regulation. On the intestinal barrier, the onion extracts did not produce toxicity and showed an antioxidant effect, which makes them interesting in the prevention of intestinal diseases related to oxidative stress, such as intestinal cancer. In silico studies have shown that the phenolic acids from onion waste extracts may have a higher intestinal permeability than its flavonoids, and being protocatechuic acid, the molecule has a higher P_app_ value. Nevertheless, even when only protocatechuic acid showed a high intestinal permeability, the other phenolic compounds may contribute to the protective role of these extracts as radical and ROS scavengers. In vitro experiments represent the first step in evaluating the health effect of new functional ingredients. Although clinical studies are necessary to prove the properties of the extract from onion waste found in this study, the results obtained in this work are open to potential use in the circular economy by obtaining new high-value ingredients for health-related products (nutraceuticals and pharmaceuticals).

## Figures and Tables

**Table 1:** Phenolic composition of extracts obtained from onion household wastes.

Phenolic Compound	Concentration (mg/g Extract)
Protocatechuic acid	11.5 ± 0.3
Ellagic acid	0.10 ± 0.01
Vanillic acid	0.33 ± 0.03
Quercetin	10.2 ± 0.4
Quercetin 3-glucoside	1.16 ± 0.08
Kaempferol	0.44 ± 0.01
Isorhamnetin	0.24 ± 0.01
Unknown flavonoid 1 ^1^	2.57 ± 0.09
Unknown flavonoid 2 ^1^	12.3 ± 0.3
Unknown flavonoid 3 ^1^	0.87 ± 0.03

^1^ Quantified as quercetin equivalents (mg/g extract).

**Table 2:** Antioxidant capacity and total phenolic and flavonoid content of the extracts obtained from onion household wastes.

Assays	Concentration
Antioxidant capacity by ABTS (mmol Trolox/g extract)	1.11 ± 0.08
Antioxidant capacity by FRAP (mmol Trolox/g extract)	0.83 ± 0.01
Antioxidant capacity by DPPH (mmol Trolox/g extract)	0.49 ± 0.08
Total Flavonoids (mg quercetin/g extract)	64 ± 3
Total Phenolic Content (mg gallic acid/g extract)	177 ± 9

**Table 3:** In-silico study of the phenolic compounds in the onion extracts.

	Identified Compound	MW	TPSA	Log P	No. Atoms	Hydrogen Bonds Acceptors	Hydrogen Bonds Donors	Rotatable Bonds	Molecular Volume (Å^3^)	Violations to LIRF	% ABS	log P_app_ (10^−6^ cm/s)
1	Protocatechuic acid	154.12	77.75	0.88	11	4	3	1	127.08	0	89.15	1.15
2	Ellagic acid	302.19	141.33	0.94	22	8	4	0	221.78	0	60.24	0.33
3	Vanillic acid	168.15	66.76	1.19	12	4	2	2	144.61	0	85.97	0.33
4	Quercetin	302.24	131.35	1.68	22	11	7	1	240.08	0	63.68	−0.23
5	Quercetin 3-glucoside	464.38	210.50	−0.36	33	12	8	4	372.21	2	36.38	0.24
6	Kaempferol	286.24	111.12	2.17	21	6	4	1	232.07	0	70.66	0.03
7	Isorhamnetin	316.26	120.36	1.99	23	7	4	2	257.61	0	67.48	−0.003
Compounds of matching spectra for the unknown flavonoids												
	Quercetin 3,7,4′-triglucoside	788.66	368.81	−4.16	55	22	14	0	636.45	3	−18.24	−1.14
	Isorhamnetin 3,4′-diglucoside	640.55	278.66	−2.07	45	17	10	8	521.86	3	12.86	−1.10
	Quercetin 4’-glucoside	464.38	210.50	−0.33	33	12	8	4	372.21	2	36.38	0.27
	Isorhamnetin 4’-glucoside	478.41	199.51	−0.03	34	12	7	5	389.73	2	40.17	0.34
	Quercetin 7,4’-diglucoside	626.52	289.65	−2.12	44	17	11	7	504.33	3	9.07	−1.22

MW = Molecular weight; TPSA = Total polar surface area; LogP = octanol-water partition coefficient, Violations to LIRF=Violations to Lipinski’s rule of five; %ABS = Theoretical absorption percentage; log P_app_ = logarithm of the apparent permeability coefficient.

## Data Availability

The data presented in this study are contained within the article and supplementary material.

## Supplementary Materials

The following are available online at https://www.mdpi.com/article/10.3390/antiox11040733/s1, Figure S1: UV spectra of the unidentified peaks of different quercetin derivatives. The upper spectrum corresponds to peak 3 (a) and the other two spectra correspond to peaks 6 (b) and 7 (c), respectively.
