# Supplementary material for: Valorization of Onion Waste by Obtaining Extracts Rich in Phenolic Compounds and Feasibility of Its Therapeutic Use on Colon Cancer
Source: Antioxidants (Basel). 2022 Apr 7;11(4):733. doi: 10.3390/antiox11040733 (PMC9032738; doi:10.3390/antiox11040733)
Supplement: Supplementary file 1 [file antioxidants-11-00733-s001.zip › antioxidants-1664511-supplementary-done.pdf]

antioxidants

MDPI

Supplementary Material

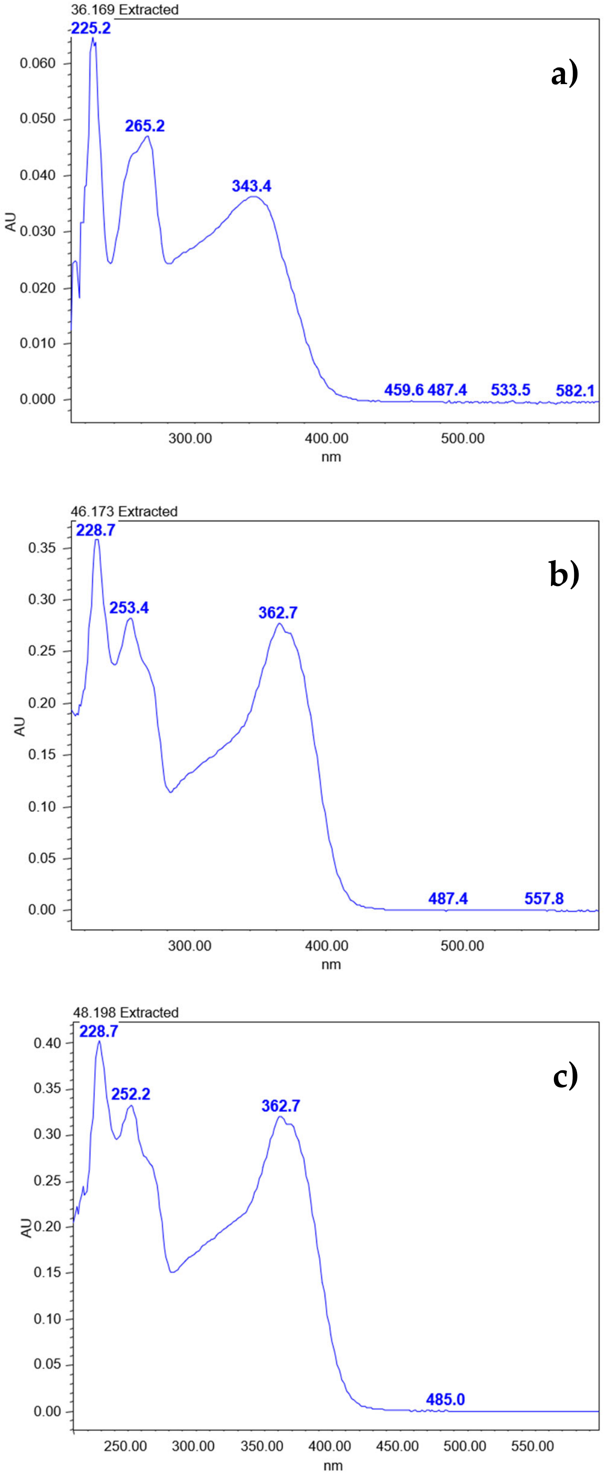

Figure S1. UV spectra of the unidentified peaks of different quercetin derivatives. The upper spectrum corresponds to peak 3 (**a**) and the other two spectra correspond to peaks 6 (**b**) and 7 (**c**), respectively.
